# Efficacy of D-Mannose Monotherapy vs. Other Agents in Preventing Recurrent Urinary Tract Infections in Women: A Systematic Review and Meta-Analysis

**DOI:** 10.5152/tud.2026.25070

**Published:** 2026-03-13

**Authors:** Ahmed Al-Hajjaj, Alaa Al- Maatoq, Asaad Al- Asadi

**Affiliations:** 1 Department of Urology, Countess of Chester Hospital, MRCS, UK; 2 Basra Medical College, MRCS, Iraq; 3 Nottingham University Hospitals NHS Trust, MRCP, UK

**Keywords:** D-mannose, Non-antibiotic prophylaxis, Recurrent UTIs, Systematic review, Urinary tract infections

## Abstract

Recurrent urinary tract infections (rUTIs) are common and often difficult to manage among women and are frequently treated with antibiotic prophylaxis. However, growing awareness of antibiotic resistance has encouraged interest in non-antibiotic alternatives such as D-mannose, a naturally occurring sugar believed to prevent rUTIs by inhibiting bacterial adhesion to the urothelial surface. This review aims to evaluate the efficacy and safety of D-mannose monotherapy in preventing rUTIs in adult women compared with antibiotics, placebo, or no treatment. A systematic review and meta-analysis were conducted following the Preferred Reporting Items for Systematic Reviews and Meta-Analyses (PRISMA) guidelines. Literature searches were performed using PubMed and EMBASE. Eligible studies were randomized controlled trials or cohort studies evaluating D-mannose monotherapy for rUTIs prevention in adult women. The primary outcome was UTI recurrence, and the secondary outcome was adverse effects. Five studies met the inclusion criteria (3 RCTs, 1 prospective cohort, and 1 retrospective cohort), including 1038 women. A meta-analysis of 3 eligible studies comparing D-mannose to placebo/control (n = 831) demonstrated a non-significant reduction in rUTIs in the D-mannose group (risk ratio = 0.37, 95% CI 0.11-1.30, P = .10) with high heterogeneity (I2 = 94%). The largest and highest-quality study showed no significant difference between D-mannose and placebo. Across all included studies, D-mannose appears to be well tolerated, with only mild and infrequent side effects. Although D-mannose appears safe and well-tolerated, the current evidence does not support its routine use in the prevention of recurrent urinary tract infections. Further high-quality trials are necessary to clarify its effectiveness in clinical practice.

**Main Points**

- This is the first systematic review and meta-analysis to specifically evaluate the use of D-mannose monotherapy for the prevention of recurrent urinary tract infections (rUTIs) in women.
- The included studies focused exclusively on D-mannose as a standalone agent, excluding combination therapies to isolate its specific effect.
- Pooled results suggested a non-significant trend toward reduced recurrence with D-mannose compared to placebo or no treatment, with considerable heterogeneity across studies.
- D-mannose was consistently reported to be safe and well tolerated, with minimal adverse effects.
- The findings highlight the need for further large-scale, high-quality randomized controlled trials to confirm the potential clinical role of D-mannose in rUTI prevention.

## Introduction

Urinary tract infections (UTIs) are among the most prevalent bacterial infections in women, with lifetime incidence estimates ranging from 40% to 50%.^1^^,2^ Approximately 20%-30% of affected women experience additional episodes, and nearly 25% go on to develop recurrent UTIs (rUTIs), defined as 2 or more infections within 6 months or 3 within a year.^3^^,^^4^ *Escherichia coli* (*E. coli*) is the primary pathogen, accounting for around 80% of all UTI cases.^5^

Traditionally, prophylactic low-dose antibiotics have been prescribed to manage rUTIs. However, long-term antibiotic use has been linked to adverse effects and rising antimicrobial resistance, highlighting the need for alternative approaches.^6^

D-mannose is a naturally occurring monosaccharide that is excreted unchanged in urine.^7^ Its proposed mechanism of action in UTI prevention is by inhibition of bacterial adhesion to the urothelial lining. D-mannose binds and blocks FimH adhesin, a protein located at the tip of type 1 pili on the bacterial surface and demonstrates a competitive inhibition against bacterial adhesion to the receptors of urothelial cells.^8^ These type-1 bacterial pili have been demonstrated on *E. coli* as well as other members of the Enterobacteriaceae family.^9^^,^^10^

Despite its widespread availability as an over-the-counter supplement, the effectiveness of D-mannose in preventing rUTIs remains controversial. Previous systematic reviews revealed mixed findings regarding the efficacy of D-mannose in preventing rUTIs, with some suggesting benefit and others highlighting insufficient evidence.^11^^,^^12^^,13^ Many of these reviews included studies using D-mannose along with other agents or focused on heterogeneous populations, which limits the ability to draw conclusions about the effectiveness of D-mannose.

In view of the persistent clinical need for effective non-antibiotic preventive options, this systematic review and meta-analysis aimed to evaluate the efficacy and safety of D-mannose when used alone (monotherapy) in preventing rUTIs in adult women compared with antibiotics, placebo, or no treatment.

## Material and Methods

### Search Strategy and Study Selection

Literature searches were conducted in PubMed and EMBASE to identify English-language studies evaluating D-mannose monotherapy in rUTI prevention, with the final search completed on November 24, 2024. The review methodology followed the PRISMA framework.^14^ Duplicate records were removed using EndNote and manually verified.

### Eligibility Criteria

A PICOS (Patient, Intervention, Comparator, Outcome, Study) structure was used for determining the study questions and the inclusion/exclusion criteria. The question was “Is D-mannose effective in the prevention of recurrent urinary tract infections in women?”

The primary outcome was the number of patients who experienced at least 1 UTI (clinically or microbiologically diagnosed) after starting D-mannose or other agents. The secondary outcome was the safety/tolerability of D-mannose.

#### Inclusion Criteria:

- Randomized controlled trials (RCTs), prospective cohort studies, or retrospective cohort studies.
- Use of D-mannose monotherapy (without other agents) for prevention of recurrent urinary tract infections.
- Comparison with antibiotics, placebo, or no treatment.
- Female participants aged ≥18 years with a history of recurrent UTIs, defined as ≥2 episodes in 6 months or ≥3 in 12 months.

#### Exclusion Criteria:

- Studies using D-mannose in combination with other agents.
- Non-human or in vitro studies.
- Articles published in languages other than English.
- Case reports or conference abstracts.

### Data Extraction

The systematic literature review was performed by 2 authors who independently identified the eligible studies for the systematic review and data extraction.

For each included study, the following data were extracted:

Author(s), year of publication, country.

Study design and duration of follow-up.

Demographics and baseline characteristics of participants.

D-mannose dosage and formulation.

Comparator details (e.g., type and dose of antibiotic, placebo/control).

Number of patients with at least 1 UTI.

Adverse events.

### Meta-Analysis

A meta-analysis was conducted to quantitatively compare the effect of D-mannose vs. other agents (antibiotics, placebo, or no treatment) in preventing rUTIs. A minimum of 3 eligible studies was required to conduct a meta-analysis to ensure statistical validity. Only studies with at least 6 months of follow-up and a comparator arm were included. A random-effects model was used to estimate pooled effect sizes. Heterogeneity was assessed using the *I*^2^ statistic.

### Quality Assessment

The quality of the included studies was evaluated using the Newcastle–Ottawa Scale if they were cohort/case-control studies.^15^ For RCTs, the Revised Cochrane risk-of-bias tool for randomized trials was employed.^16^

## Results

### Literature Search and Study Selection

**Figure 1.**
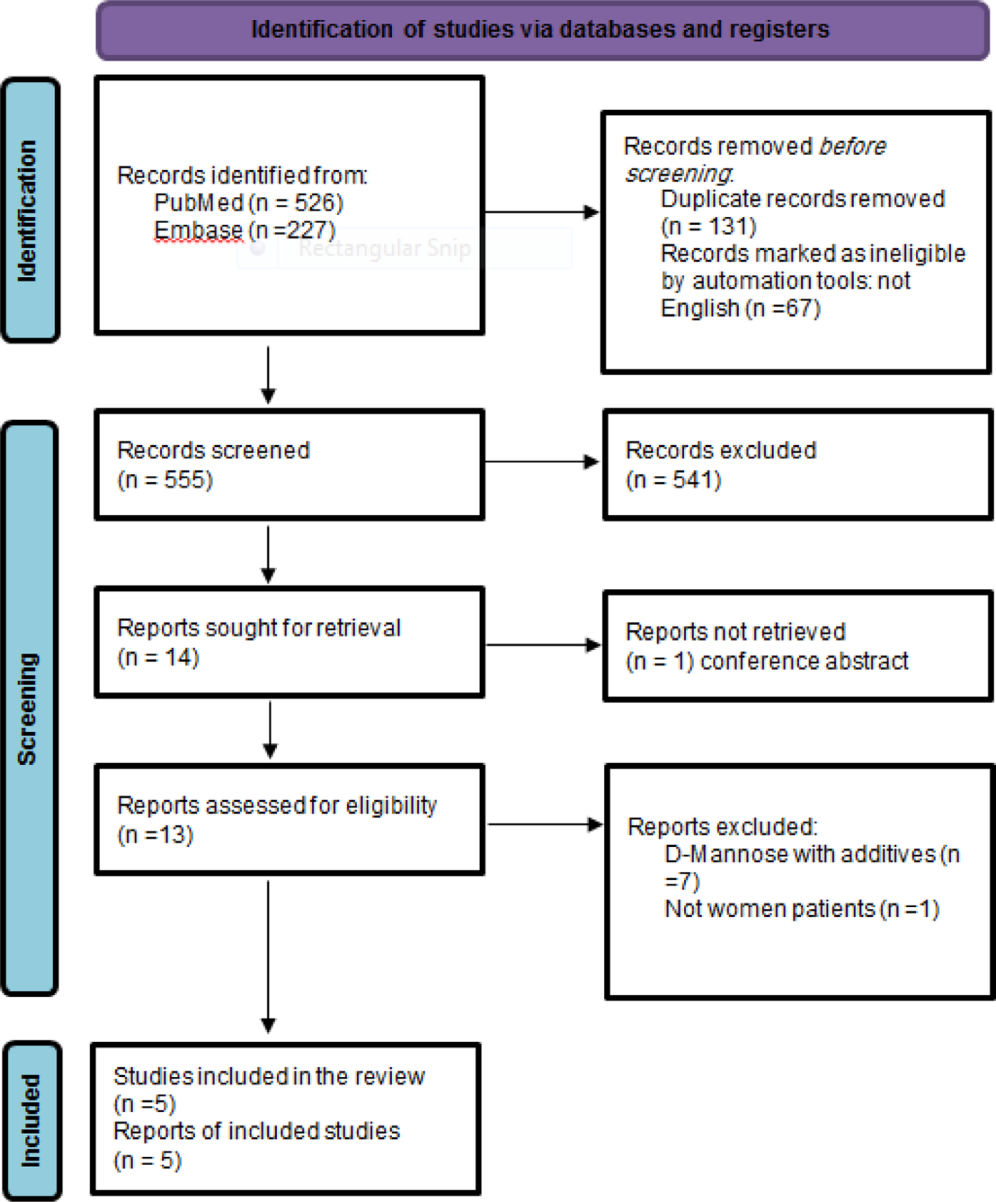
PRISMA flow diagram showing the study selection process.

A total of 753 unique citations were screened, with 13 full texts reviewed. Five studies satisfied all inclusion criteria. The study selection process is detailed in the PRISMA flowchart (Figure 1).

### Characteristics of the Included Studies

**Figure 2.**
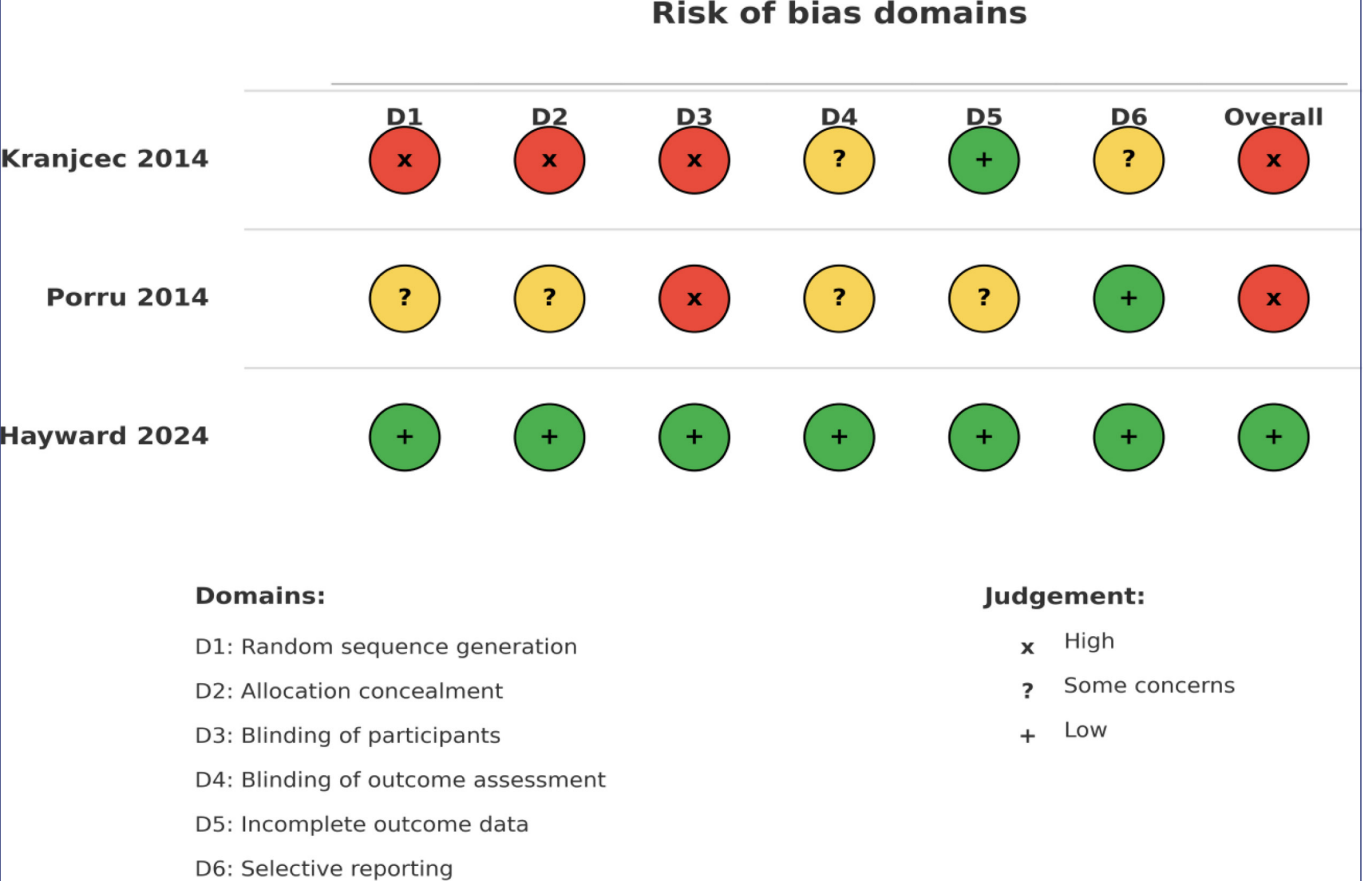
Risks of Bias Assessment (RoB-1).

Out of the 5 included studies, 3 were RCTs, 1 was a prospective cohort, and 1 was a retrospective cohort study. A total of 1038 women were included, with the largest study enrolling 598 participants and the smallest enrolling 27 participants. Methodological characteristics are outlined in Table 1, and key findings from each study are summarized in Table 2. Table 3 provides the risk of bias and evidence grading for RCTs while Table 4 summarizes the quality assessment of cohort studies. A visual summary of the risk-of-bias assessment for RCTs is presented in Figure 2.

### Individual Study Summaries

Kranjcec: This trial compared D-mannose, nitrofurantoin, and no treatment in 308 women.^17^ Over 6 months, recurrent UTI rates were significantly higher in the no prophylaxis group (60.8%) compared to D-mannose (14.6%) and nitrofurantoin groups (20.4%; *P* < .001). The main limitation of the study was a high risk of bias due to the open-label design.

Porru conducted an open-label randomized crossover trial with 60 women comparing D-mannose to intermittent trimethoprim/sulfamethoxazole prophylaxis.^18^ Recurrence occurred in 75% of antibiotic-treated women compared to 20% in the D-mannose group over 24 weeks. Limitations included intermittent antibiotic dosing and the lack of a washout period.

Domenici performed a prospective cohort study involving 45 participants randomized to prophylaxis with D-mannose or no prophylaxis after initial cystitis treatment.^19^ Recurrence was significantly lower in the D-mannose group (4.5%) compared to controls (33.3%; *P* = .05). The main limitations were a small sample size and intermittent D-mannose dosing.

Chiu conducted a retrospective cohort analysis of 27 postmenopausal women.^20^ UTI incidence rates significantly decreased after initiating D-mannose, particularly among women with cystitis cystica lesions. Limitations included retrospective design, small sample size, and lack of detailed recurrence data, precluding inclusion in meta-analysis.

Hayward performed the largest double-blind randomized placebo-controlled trial (n = 598).^21^ Recurrence rates were not significantly different between D-mannose (51.0%) and placebo groups (55.7%; risk ratio (RR) 0.92, 95% CI 0.80-1.05). Limitations included reliance on clinical diagnosis without microbiological confirmation. This high-quality study raises questions about the clinical relevance of findings from smaller and less rigorous trials.

### Excluded Studies

Studies excluded from this review primarily used combination therapies involving D-mannose with other agents. For example, Palleschi 2017,^22^ Russo 2019,^23^ Kuzmenko 2019,^24^ and De Leo 2017^25^ combined D-mannose with supplements such as NAC, cranberry, or propolis extracts. Additional studies (Rau 2024,^26^ Phe 2017,^27^ Sergio Venturini 2024,^28^ and Lenger 2023^29^) were excluded due to methodological issues such as mixed patient populations, early termination, or combination interventions. These exclusions helped to maintain a clear focus on the effect of D-Mannose alone.

### Meta-Analysis of D-Mannose vs. Placebo/Control

**Figure 3.**
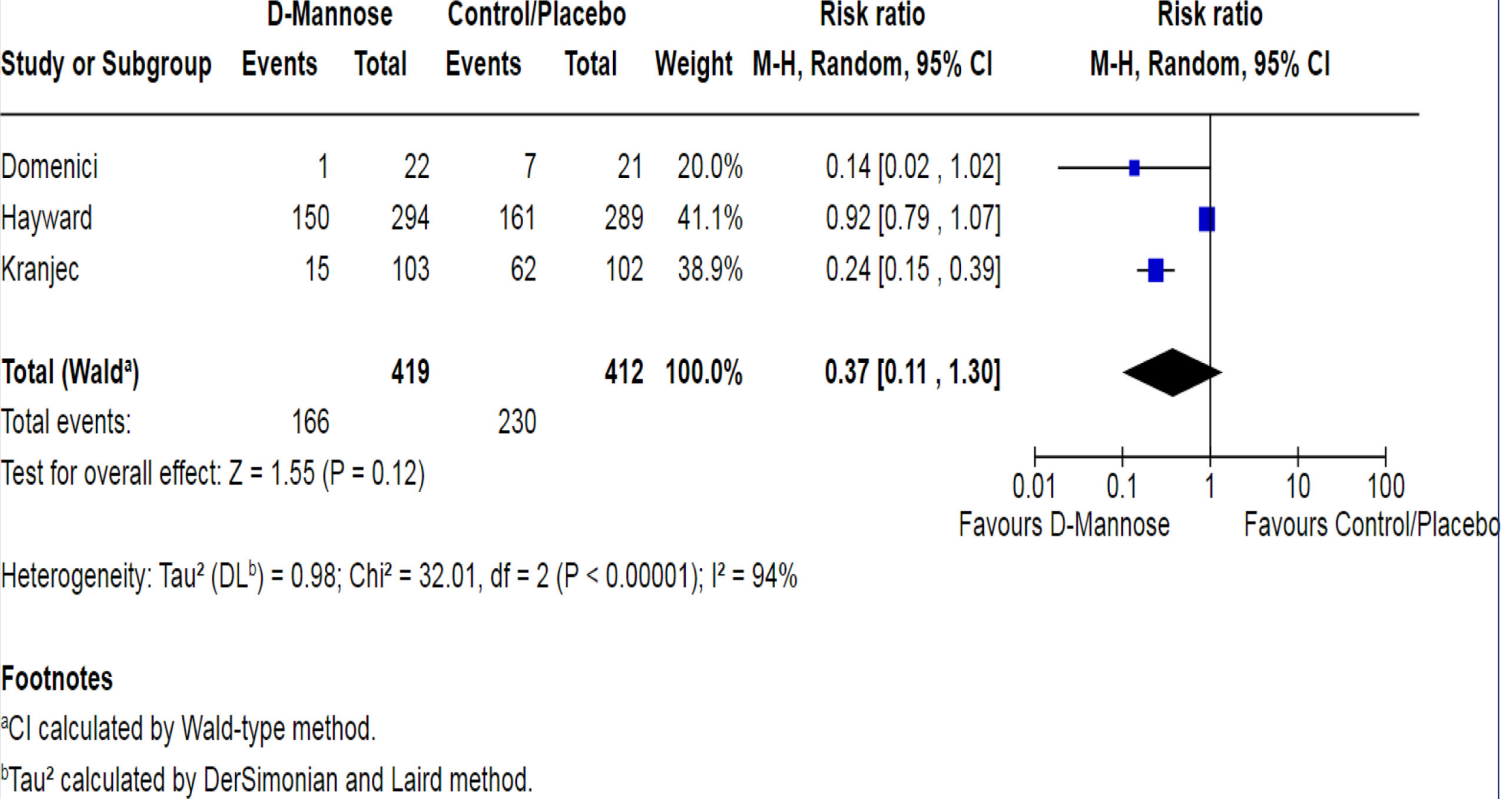
Forest plot of pooled risk ratios comparing D-mannose vs. placebo/control in prevention of recurrent urinary tract infections in women. Random-effects model used.

A meta-analysis was performed on 3 studies (Domenici,^19^ Hayward,^21^ and Kranjcec^17^) that met the inclusion criteria for quantitative assessment (Figure 3). Across these 3 trials, a total of 419 women in the D-mannose group and 412 women in the control/placebo group were analyzed. The pooled RR was 0.37 with a wide 95% CI (95% CI, 0.11-1.30; *P* = .12), suggesting a non-significant trend toward fewer recurrent UTIs in the D-mannose group compared to control/placebo. There was substantial heterogeneity among the included studies (*I*^2^ = 94%, *P* < .00001). Hence, a random-effects model using the Mantel-Haenszel method was applied.

Two smaller studies (Domenici and Kranjcec) demonstrated a large effect size in favor of D-Mannose (RR = 0.14 and 0.24, respectively). However, the largest trial by Hayward et al^21^ (n = 598) showed a minimal effect (RR = 0.92, 95% CI, 0.78-1.09). This discrepancy between the large, high-quality randomized controlled trial (RCT) and the smaller studies contributed significantly to the overall heterogeneity. Despite the point estimate suggesting a protective effect of D-Mannose, the wide CI crossing 1 and the non-significant *P*-value indicate that, when all 3 studies are combined, the overall benefit cannot be established. Further large-scale, standardized RCTs with consistent dosing and outcome measures are needed to clarify the efficacy of D-Mannose in preventing recurrent UTIs.

### Heterogeneity and Sensitivity Analysis

As noted above, the meta-analysis showed substantial heterogeneity. To explore potential sources of this heterogeneity, differences were examined in study design, sample size, diagnostic criteria, and dosing regimens. Notably, the 2 smaller, open-label trials (Kranjčec^17^ and Domenici^19^) showed a pronounced effect in favor of D-mannose, whereas the largest, double-blind placebo-controlled RCT (Hayward^21^) showed no significant difference between intervention and control groups. These differences in methodology and population characteristics likely contributed to the high *I*^2^ value. When smaller, open-label studies were considered separately from the larger blinded RCT, the direction of effect remained similar, but the precision and certainty of the pooled estimate decreased. This pattern suggests that the overall findings are influenced predominantly by study design and size, underscoring the importance of larger, methodologically rigorous trials to clarify the true effect of D-mannose.

### Adverse Events

D-mannose was generally well tolerated across all studies, with no serious adverse events (SAEs) attributed to the intervention. In Kranjčec,^17^ 8/103 participants (7.8%) reported mild diarrhea compared with 29/103 (27.2%) in the nitrofurantoin group (RR 0.276; 95% CI 0.132-0.574; *P* < .0001). Porru^18^ and Domenici^19^ reported no clinically significant side effects. In Hayward 2024, 20 SAEs occurred in the D-mannose arm and 8 in the placebo arm, none treatment-related, and no specific non-serious AE pattern was noted. Chiu 2022 did not report any adverse event data. A summary of adverse events by study is provided in Table 5.

## Discussion

### Principal Findings

The systematic review and meta-analysis assessed the efficacy of D-mannose monotherapy for the prevention of rUTIs in women. While smaller studies suggested a potential reduction of recurrence, the largest and highest quality RCT (Hayward et al^21^) to date reported no statistically significant difference compared to placebo. The Hayward trial used clinical diagnosis alone, reflecting a pragmatic real-world approach that enhances generalizability. However, this broader definition may also have included some participants without microbiologically confirmed infection, which could partly explain the smaller observed treatment effect compared with earlier, more narrowly defined studies.

Among the 3 studies included in the meta-analysis, the pooled (RR = 0.37; 95% CI: 0.11-1.30; *P* = .12) indicated a trend toward reduced UTI recurrence with D-mannose compared to placebo or no treatment, but the substantial heterogeneity observed (*I*^2^ = 94%) limits the certainty of this effect. The observed variability likely reflects differences in study design, population characteristics, outcome definitions, and D-mannose dosing regimens. As the more promising results tended to come from smaller, open-label studies with methodological limitations, this casts doubt on how applicable their findings are to a broader clinical practice.

D-mannose appears to be consistently well tolerated across all included studies, with few reported side effects. However, the current evidence does not support the routine use of D-mannose monotherapy for rUTI prevention in clinical settings. It may nevertheless be considered in selected subgroups—such as postmenopausal women or patients who are unfit for or prefer to avoid antibiotic prophylaxis—provided they are counseled regarding the limited and inconclusive nature of the current evidence.

### Comparison with Previous Studies

The findings are in agreement with the Cochrane review by Cooper et al (2022),^12^ which found insufficient evidence to recommend D-mannose as an effective monotherapy for rUTI prevention. Cooper and colleagues highlighted the need for additional high-quality randomized trials due to inconsistencies and methodological weaknesses in the existing literature. In contrast, Lenger et al (2020)^11^ suggested that D-mannose may be comparable to antibiotic prophylaxis, and Han et al (2024)^13^ reported a beneficial effect of D-mannose in their network meta-analysis. These more optimistic conclusions are likely attributable to broader inclusion criteria in those reviews, which assessed D-mannose both as monotherapy and in combination with other agents (e.g., probiotics, cranberry), and relied heavily on small, open-label studies. By focusing exclusively on D-mannose monotherapy and incorporating the recent, large, high-quality RCT by Hayward et al^21^, the review provides a more stringent and clinically focused evaluation. The absence of a statistically significant treatment effect and the null results from the Hayward trial indicate that D-mannose, while safe, cannot currently be recommended as an alternative to antibiotic prophylaxis for rUTI prevention. As such, the review reflects the most contemporary evidence base and offers the clearest estimate to date of the isolated effect of D-mannose monotherapy.

### Strengths and Limitations

To current knowledge, this is the first systematic review and meta-analysis that specifically examines D-mannose as a standalone intervention for recurrent UTI prevention in women, incorporating the most recent and high-quality clinical trials.

Key strengths include well-defined inclusion criteria, adherence to PRISMA guidelines, and comprehensive search across major databases. Independent review by 2 authors and the use of validated quality assessment tools further strengthened the methodological rigor. However, several limitations should be acknowledged. The number of eligible studies was small, and effect estimates were driven largely by 1 large RCT. Many of the earlier trials were small, open-label studies with variable dosing regimens and heterogeneous outcome definitions, which may introduce performance and detection bias and contribute to the observed heterogeneity. In addition, only English-language studies were included. These factors should be considered when interpreting the findings, as they limit the certainty and generalizability of the results.

### Clinical Relevance

Given the current evidence, D-mannose cannot be recommended as a standard prophylactic option for recurrent urinary tract infections. That said, it may still be considered in women who seek non-antibiotic alternatives or who are not candidates for antibiotic prophylaxis, provided they receive appropriate counseling on its limited and uncertain benefits.

### Recommendations for Research

There is a clear need for further larger, well-designed RCTs to define the role of D-mannose. Standardized dosing, clearly defined and microbiologically confirmed outcomes, and extended follow-up periods would improve the quality of future evidence.

## Conclusion

Although D-mannose is safe and well tolerated, it cannot yet be recommended as a standard prophylactic option in preventing recurrent urinary tract infections in women. Further high-quality randomized trials are needed.

## Figures and Tables

**Table 1.** Characteristics of the Included Studies

**Authors**	**Study Design**	**Study Population**	**Inclusion Criteria **	**Exclusion Criteria**	**D-mannose Group (Dose, Duration)**	**Compared Group**
Kranjcec^17^	Randomized controlled trial	Women age ≥18 years (median 48-52, range 29-58) n = 308	Acute cystitis and history of recurrent UTIs	Pregnant/breastfeeding symptoms of pyelonephritis, urinary tract anomalies, diabetes, current hormone therapy/contraception, previous antibiotic prophylaxis	Prophylaxis: 2 g of oral D-mannose powder daily × 6 months (n = 103)	Active prophylaxis: 50 mg of Nitrofurantoin daily × 6 months (n = 103) or Control prophylaxis: none(n = 102)
Porru^18^	Randomized cross-over trial	Women age ≥18 years (median age 42 years, range 22-54) n = 60	Current acute symptomatic UTI and history of recurrent UTIs	Symptoms of pyelonephritis, renal disease, anatomical abnormalities, prior gynaecological surgery, immunosuppressive medications or diseases, pregnant/breastfeeding	Oral D-mannose 1 g TID Prophylaxis: TID × 2 weeks then BID × total 6 months (n = 30). Cross-over to other group for additional 6 months	Oral TMP/SMX 160/800 mg BID × 5 days Prophylaxis: TMP/SMX 160 mg/800 daily (1 week each month) × total 6 months (n = 30). Cross-over to other group for additional 6 months
Domenici^19^	Prospective cohort	Women, age 18-65 years (mean 46.7 ± 5.7 years) n = 45	Acute cystitis and/or history of recurrent UTIs	urinary tract anomalies, pregnancy/breastfeeding, symptoms of pyelonephritis, upper tract infection, hormone therapy, diabetes, use of CISC, previous antibiotic prophylaxis	Prophylaxis: oral Mannocist daily (1 week every other month) × 6 months (n = 22)	Prophylaxis: None (n = 21)
Chiu^20^	Retrospective cohort	Women age ≥18 years (median age 73 years, range 67-79) n = 27	Women with rUTIs who had a cystoscopy performed for history of refractory rUTIs, were taking D-mannose for UTI prevention and had at least 1 year of follow-up both before and after initiating D-mannose	any patient who is taking vaginal oestrogen or methenamine	One gram twice daily or 2 grams once daily for 12 months (n = 27).	Prophylaxis: None (n = 27). No prophylaxis for 12 months prior to D-mannose use.
Hayward^21^	Multi-centre double-blind randomized placebo-controlled trial	Women age ≥18 years (median age 58 years, range 19-93) n = 598	Women with symptoms consistent with a UTI and/or resulting in a UTI-specific antibiotic prescription 3 or more times in the past year or 2 or more times in the past 6 months	Pregnant, lactating, or planning pregnancy during the study; history of interstitial cystitis or overactive bladder syndrome; were a nursing home resident; catheterized, including intermittent self-catheterization, or had participated in a research study involving an investigational medicinal product in the past 12 weeks, had started prophylactic antibiotics in the past 3 months or intended to start them during the next 6 months or were currently using D-mannose	Daily scoop amounting to approximately 2 g of D-mannose powder or a similar for total of 6 months (n = 294)	Placebo: daily scoop of fructose powder for total 6 months. (n = 289)

UTI, urinary tract infection; TMP/SMX, trimethoprim-sulfamethoxazole; CISC, clean intermittent self-catheterization.

**Table 2.** Summary of the Findings

**Authors**	Number of rUTIs* in D-Mannose Group (%)	**Number of rUTIs in Antibiotics Group (%)**	**Number of rUTIs in Control/Placebo Group (%) **
Kranjcec^17^	15/103 (14.6)	21/103 (20.4)	62/102 (60.8)
Porru^18^	12/60 (20)	45/60 (75)	N/A
Domenici^19^	1/22 (4.5)	N/A	7/21 (33.3)
Chiu^20^	N/A	N/A	N/A
Hayward^21^	150/294 (51.0)	N/A	161/289 (55.7)

rUTIs, recurrent urinary tract infection.

*Number of patients experiencing at least one recurrent urinary tract infection during follow-up.

**Table 3.** Summary of Risk Bias Assessment and Grade of Evidence of Included Clinical Trials

**Authors**	**Random Sequence Generation**	**Allocation Concealment**	**Blinding of Participants and Personnel**	**Blinding of Outcome Assessment**	**Incomplete Outcome Data**	**Selective Reporting**	**Grade of Evidence**
Kranjcec^17^	↑	↑	↑	Unclear	↓	Unclear	Fair
Porru^18^	Unclear	Unclear	↑	Unclear	Unclear	↓	Fair
Hayward^21^	↓	↓	↓	↓	↓	↓	Good

**Table 4.** Summary of the Quality Assessment of the Included Cohort Studies

**Author**	**Question #**	**Selection**	**Question #**	**Comparability**	**Question #**	**Outcome**	**Study Quality**
Domenici^19^	1	*	1	—	1	—	6/9
	2	*	2	—	2	*	
	3	*			3	*	
	4	*					
Chiu^20^	1	—	1	*	1	*	6/9
	2	*	2	—	2	*	
	3	*			3	—	
	4	*					

*Indicates that the criterion was fulfilled according to the Newcastle–Ottawa Scale.

**Table 5.** Reported Adverse Events by Study

**Study**	**D-Mannose Group (n)**	**AE Reported**	**SAE Related to Treatment**
Kranjcec^17^	103	8 (7.8%) - diarrhea only	0
Porru^18^	30	Not quantified; no significant side effects	Not reported
Domenici^19^	22	No side effects reported	Not reported
Hayward^21^	294	20 SAEs (none treatment related); no AE pattern	0
Chiu^20^	—	Not reported	—

—, not reported; AE, adverse event; SAE, serious adverse event.

## Data Availability

The data that support the findings of this study are available on request from the corresponding author.
